# The effectiveness of "Exercise on Prescription" in stimulating physical activity among women in ethnic minority groups in the Netherlands: protocol for a randomized controlled trial

**DOI:** 10.1186/1471-2458-8-406

**Published:** 2008-12-10

**Authors:** Karen Hosper, Marije Deutekom, Prof Karien Stronks

**Affiliations:** 1 Department of Social Medicine, Academic Medical Centre, Amsterdam, the Netherlands

## Abstract

**Background:**

Lack of physical activity is an important risk factor for overweight, diabetes, cardiovascular disease and other chronic conditions. In the Netherlands, ethnic minority groups are generally less physically active and rate their own health poorer compared to ethnic Dutch. This applies in particular to women. For this reason women from ethnic minority groups are an important target group for interventions to promote physical activity.

In the Netherlands, an exercise referral program ("Exercise on Prescription") seems successful in reaching women from ethnic minority groups, in particular because of referral by the general practitioner and because the program fits well with the needs of these women. However, the effect of the intervention on the level of physical activity and related health outcomes has not been formally evaluated within this population. This paper describes the study design for the evaluation of the effect of "Exercise on Prescription" on level of physical activity and related health outcomes.

**Methods:**

The randomized controlled trial will include 360 inactive women from ethnic minority groups, with the majority having a non-Western background, aged between 18 and 65 years old, with regular visits to their general practitioner. Participants will be recruited at healthcare centres within a deprived neighbourhood in the city of The Hague, the Netherlands.

An intervention group of 180 women will participate in an exercise program with weekly exercise sessions during 20 weeks. The control group (n = 180) will be offered care as usual. Measurements will take place at baseline, and after 6 and 12 months. Main outcome measure is minutes of self reported physical activity per week. Secondary outcomes are the mediating motivational factors regarding physical activity, subjective and objective health outcomes (including wellbeing, perceived health, fitness and body size) and use of (primary) health care. Attendance and attrition during the program will be determined.

**Conclusion:**

This trial will provide information on the effectiveness of an exercise referral scheme on the short and long term among women from ethnic minority groups, mainly non-Western, in the Netherlands. The results of this study will contribute to the evidence base for interventions in ethnic minority populations.

**Trial registration:**

Dutch Trial register: NTR1294

## Background

Regular physical activity plays an important role in the prevention and treatment of overweight, diabetes, cardiovascular disease and other chronic conditions [1-5]. In the Netherlands, only half of the population meets the current recommendations for physical activity [1]. Several studies have shown that ethnic minority populations in the Netherlands are even less physically active than the ethnic Dutch population [1,6]. This lack of physical activity may be one of the factors explaining disparities in health. For example, Turkish and Moroccan women are more often overweight (respectively 72% and 64%) than ethnic Dutch women (45%) [1]. In addition, Turkish, Moroccan and Surinamese women more often suffer from diabetes mellitus, hypertension, and mental health problems than Dutch women in the same age group and rate their own health poorer [7-10].

For this reason women from ethnic minority groups are an important target group for interventions to promote physical activity. Of the interventions that have been developed in the Netherlands over the past years, "Exercise on Prescription" is one that seems successful in reaching ethnic minority populations [11,12]. Interventions such as "Exercise on Prescription" are internationally referred to as exercise referral schemes (ERS) [13,14]. In ERS, patients who are physically inactive or have related health problems are referred by their general practitioner to supervised exercise activities. Trained instructors collaborate with the general practitioners to support the ongoing rehabilitation of those referred. There have been several studies that evaluated the effect of ERS among the general population [13,15-18]. These studies generally indicated that ERS have a small but positive effect on increasing physical activity in sedentary people. However, to date no study has examined the effect of ERS in promoting physical activity on participants from varying ethnic minority groups.

A process evaluation of the Dutch program "Exercise on Prescription" (EoP) showed that it successfully reaches women from ethnic minority groups, mostly with a Surinamese, Turkish and Moroccan background [19]. Qualitative interviews among participants showed that EoP is attractive for these women because of the facilitating role of the health professional, the supportive environment, a financial incentive (in the form of a discount after successful completion of the program), supervision by fitness instructors, the proximity of locations and the possibility of 'women-only' exercise groups [19]. Women also indicated in the interviews that they had become more physically active and that their (subjective) health and psychosocial well- being had improved. Moreover, 74% said that they intended to keep exercising after participation in EoP ended [11]. While these experiences with EoP seem very positive, the effect of the intervention on the level of physical activity, related health (problems) and use of health care has not been formally evaluated. The ongoing program of EoP in the city of The Hague, The Netherlands offers an interesting opportunity to study these effects among ethnic minority groups.

Therefore the objective of our study is to evaluate the effect of EoP on physical activity among women with mainly a non-Western ethnic background. Based on social cognitive models, the assumption is that this exercise intervention will finally result in a behaviour change via a positive influence on the intention and attitudes towards physical activity [20]. Therefore, we will also evaluate the effects on the intention and motivational factors with regard to physical activity (attitudes, social influence and self-efficacy). It is expected that increased physical activity will positively affect objective health (fitness, body size), subjective health (perceived health and well-being) and use of health care. Therefore also these outcome measures will be assessed additionally to the level of physical activity. Finally, attendance and attrition during the program will be determined.

## Methods

### Objectives

The present study aims at evaluating the effect of EoP, an exercise referral scheme, on the level of physical activity of women from ethnic minority groups in the Netherlands. In addition, the effect of this intervention on mediating motivational factors, health and use of health care will be evaluated. The study addresses the following questions:

1) What is the effect of participation in EoP on the level of physical activity as measured by self-reported physical activity (both immediately after the programme and six months after completion of the programme)?

2) What is the effect of participation in EoP on the intention and motivational factors with regard to physical activity (e.g. attitudes, social influences and self-efficacy)?

3) What is the effect of participation in EoP on the subjective health status of participants (perceived health and well-being) and objective elements of health (fitness of the participants and body size)?

4) What is the effect of participation in EoP on the use of (primary) health care?

Furthermore, the participation and the follow-up rate in the study (and differences between relevant sub-groups) will be assessed as well as the reasons for non-participation or loss to follow-up.

### Study Design

The study will be set up as a randomized controlled trial comparing referral to an exercise program (Exercise on Prescription (EoP)) and usual care. To evaluate the effect of the intervention it is important that we follow the referral procedure as it is currently carried out by the general practitioners. Therefore, in this study we will use pre-randomisation, which refers to a procedure in which participants are randomly allocated to the control or the intervention group without first obtaining informed consent. Whenever a women allocated to the intervention group visits her GP, she will be referred to EoP if she meets the criteria (ethnic minority background, aged 18–65 years, physically inactive). The women will be asked for informed consent after the referral. If the patient is allocated to the control group the GP will not refer the patient to EoP but provide care as usual which includes lifestyle advices regarding physical activity when necessary. This implies that the control group is not informed about the intervention or the purpose of the study as this knowledge may lead to attrition of controls, because they may prefer participating in the exercise intervention and therefore not participate in the study. As a result the control group would not be a random selection and this may lead to less valid study results. This procedure of pre-randomisation is commonly accepted in situations where knowledge of the intervention (and the study's aim) can influence the study results [21].

### Randomisation

We will use a concealed procedure for randomisation of women using a computer generated randomisation list developed by an independent person.

### Ethical Considerations

This study is approved by the Medical Ethics Committee of Academic Medical Centre of Amsterdam, and is registered within the Dutch Trial Register (NTR1294).

### Participants and Recruitment

In total, 360 women will be recruited for the study. Eligible are women, aged 18 to 65 years from ethnic minority groups. In the Netherlands, data on country of birth is considered to be an objective and stable indicator of ethnic background [22]. Unfortunately, these data were not available in the registries of GP's. Instead, we assessed ethnicity based on patient's last names in combination with the knowledge of the general practitioners on the ethnic background of their patients. Further criteria for inclusion are insufficient physical activity (e.g. not meeting the Dutch guideline for physical activity (exercising 30 minutes a day for at least 5 days a week), regular GP visits, defined as two or more visits in the past 3 months, and GP confirmed absence of a psychological or physical condition which prevents participation in the exercise programme.

Exclusion criteria are sufficient physical activity, participation in EoP in the year preceding the start of inclusion, pregnancy, diagnosis or treatment of a disorder that makes physical activity impossible or planned emigration or a long-term stay abroad.

We will recruit general practices situated in deprived neighbourhoods in The Hague in the Netherlands. The researchers will negotiate with every participating general practice the number of patients that they are willing to include in the study. Based on that number a random sample of women from ethnic minority groups will be drawn. That sample will be larger than the actual number needed to accommodate for losses resulting from the further inclusion and exclusion criteria and the possibility for women to decline participation.

The selected women in the control group will receive a letter from their General Practitioner (GP) asking their permission to be contacted by the researchers for participation in the study. The study will be introduced as a study on the health and lifestyle of women in The Hague in order to make recommendations as to how to improve the health and lifestyle of these women. The selected women can decline further participation by sending back a short reply-slip or by making a call to the research-assistant. All participants who do not actively withdraw, will be contacted by an interviewer in order to make an appointment for the interview.

Participants in the intervention group also receive a letter from their GP in which they are invited to visit their GP to discuss participation in EoP. Participants who are referred to EoP will be informed about the study and asked for their participation following the intake at EoP. They will receive similar information on the purpose of the study as the control participants. In this way we hope to avoid bias in the results as a consequence of social desirable answers (or behaviour) due to the fact that people are aware of the study's purpose. All written material such as letters and leaflets will be available in Dutch and Turkish. Given the demographics of the concerning deprived neighbourhoods in The Hague it is expected that the largest group within our study population will have a Surinamese, Turkish or Moroccan background. The main language used among Moroccan people in the Netherlands is Berber, but this is not a written language. Therefore, we could not translate the materials for Moroccan women. Surinamese people generally speak the Dutch language fluently.

### Intervention

The general practitioner will offer participation in Exercise on Prescription (EoP) to every eligible woman in the intervention group. EoP programme is financed by the municipality of The Hague and several health insurance companies. The programme is set up to promote physical activity among inhabitants of deprived neighbourhoods in The Hague by letting them experience the beneficial effects of sports and to stimulate them in such a way that they are able to continue the behaviour after completion of the programme. The main focus of the programme is on increasing participation, empowerment of the client, providing tailored activities, and increasing motivation through personal coaching. All these factors have been shown to be important parameters of behavioural changes [23].

#### Intake

Over the past years a protocol for EoP has been developed and tested in practice. This protocol consists of an intake followed by 20 sessions of supervised physical activity and a final evaluation. During the intake, the lifestyle advisor will document the individual goals and preferences with regard to physical activity and organise the participation in the exercise activities.

#### Supervised activities

After the intake, participants will be contacted again when the next available exercise course starts. At the start of the course, participants pay a contribution of 50 euros. Of these 50 euros 10 will be returned after successful completion of the programme, i.e. at least 85% attendance. Attendance is monitored by the lifestyle advisors, who are instructed to immediately contact a participant whenever she misses a session. This is considered an important part of the motivation strategy and will therefore also be part of the strategy employed in the study. At present, EoP offers Fitness, Aquarobics, Aerobics and Dancing [24]. In order to promote participation, all important issues that became evident in an earlier process evaluation of EoP will be taken into account [11]. For example, as part of the programme both daytime and evening group exercise sessions will be offered in groups accessible to women only. The timing will be such that the courses do not coincide with school times. Moreover, the courses will be organised at several locations throughout the city that are easily accessible by public transport, with a focus on those neighbourhoods with large ethnic minority populations. The physical activity component of the program is aimed at increasing cardiovascular fitness and muscle power.

The personal coaching during EoP is organised in two parts. The first ten supervised sessions are aimed at motivating the participant through increasing awareness of the positive effects of exercise. The following ten sessions are dedicated to empowering the participant with respect to the continuation of the healthy (physical) behaviour. During the sessions the participants are given individual advice as to how to reach the goal of exercising 30 minutes a day for at least 5 days a week, either by increasing daily physical activity (walking etc.) or by further participating in (un)organised sports. In addition, experienced social support, attitudes towards sports and ways of coping with (negative) feedback from community will be addressed. This supervision and coaching is done by specially trained sports instructors, under the responsibility of the lifestyle advisor.

#### Final evaluation with lifestyle advisor and further referral

After the first 20 weeks, a final evaluation takes place with the lifestyle advisor in which the individual's achievements, further goals and experiences in the programme are evaluated. At the same time, the lifestyle advisor will refer the participants to "Exercise without Prescription" [25]. Exercise without Prescription was designed to address a further aspect that was deemed important by participants in the process evaluation of EoP, namely that participants experienced difficulty in continuing the desired behaviour after EoP. In Exercise without Prescription participants are guided further through the transition of supervised activity to independent activity, through a motivational counselling session in which the participant is stimulated to continue and actively plan healthy behaviour. This is supplemented with regular invitations for free try-out lessons activities at local clubs, fitness centres or community centres. Moreover, an important part of the Exercise without Prescription programme is the possibility of a discounted membership at a fitness centre and several local facilities.

### Control Group

Women in the control group will receive care as usual and will only be approached by a professional interviewer for an interview on lifestyle (in their preferred language if possible) and measurements. Participating women will receive a voucher of 10 euro to stimulate participation in the study (participants in the intervention group will receive this voucher as well).

### Outcome Measures

The primary outcome measure is self-reported physical activity measured in minutes per week. Secondary outcome measures are a) the intention and motivational factors with regard to physical activity (e.g. attitudes, social influences and self-efficacy) b) subjective health outcomes (perceived health and well-being) c) objective health outcomes: fitness of the participants (oxygen uptake) and body size (body mass index, waist-hip ratio and waist circumference, and fat percentage) d) the use of (primary) health care. Furthermore, the participation and the follow-up rate in the study will be assessed as well as the reasons for non-participation or loss to follow-up. Baseline demographic characteristics, such as age, ethnicity, neighbourhood of residence and other socioeconomic factors of potential participants will be recorded.

Attendance during the intake, participation in the weekly sports sessions, and the final evaluation (intervention arm) will be monitored by the lifestyle advisor, who will also notify women prior to the appointment and follow-up with women who have not shown up for a session. In addition, data on the individually tailored content of the intervention and subsequent participation in "Exercise without Prescription" will be registered.

### Data Collection

**Figure 1:**
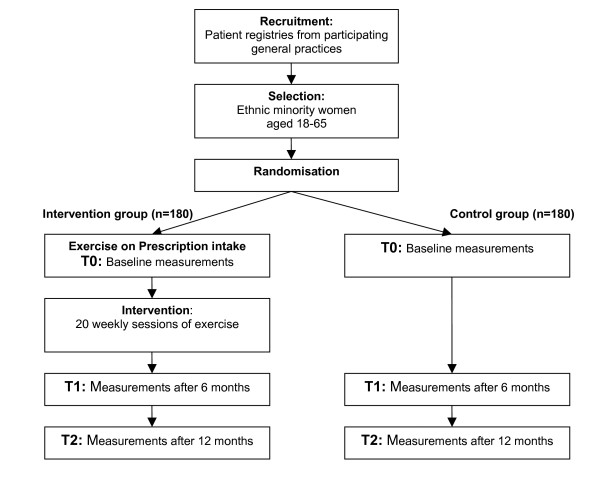
Study design.

Data collection will take place at baseline (T0, directly following the EoP -intake), after 6 months (T1 shortly after completion of EoP) and after 12 months (T2, follow-up measurement 6 months after completion of EoP) for both the intervention group and the control group (see Figure 1). All interviews and physical examinations will take place in one of the nearby health care centres that participate in the study or, when possible, in case of intervention women at the same location as the meetings with lifestyle advisor.

#### Interview

Data on self-reported physical activity and secondary outcome measures will be collected during a structured interview, which will be conducted by a trained female interviewer who has a Surinamese, Moroccan or Turkish background. The interviewers are bilingual and can address the questions in the preferred language of the participant. Whenever possible it is tried to send the same interviewer to the participants at all three time points.

During the interview a questionnaire will be used which is constructed to measure self-reported physical activity, motivational factors, subjective and objective health, and health care. Where possible, standard validated questionnaires that have been used previously among non-Western populations will be used [3,10]:

- Self reported physical activity related to travel, house-keeping, work, leisure time, and sports will be measured with the SQUASH, which has been used previously among the Turkish, Moroccan and Surinamese population [3,6,10,26].

- Questionnaires developed for a multi-ethnic population will be used to measure motivational factors related to physical activity: attitudes, social influences, self-efficacy and intention [27-29].

- Perceived health is measured with one item on self-perceived general health with a 5-point likert scale ranging from very good to very poor.

- Common physical complaints are measured such as diabetes, COPD, cardiovascular diseases with the possibility to mention other complaints.

- Well being will be measured with the W-BQ12 (well-being questionnaire)[30].

#### Physical examination (objective health measures)

The physical examination in the intervention and the control group will consist of a standardised measurement of the weight, length, percentage fat, waist and hip circumference. For these measurements the protocol of the SUNSET study will be used [10]. The physical fitness (maximal oxygen uptake) of the women will be monitored through the Siconolfi step test [31]. Briefly, patients will be asked to step up and down a portable bench for three minutes at a rate of 17 steps per minute, which will be kept constant with the help of a metronome. Heart rate will monitored continuously during the test and will recorded at the end of the stage. If target heart rate (65% of the predicted [220 minus age] maximum heart rate) is reached, the protocol will be terminated. Otherwise, a second stage (26 steps per minute) and, eventually, a third stage (34 steps per minute) will be completed with 1 minute of rest between each stage. The steady-state absolute maximal oxygen uptake can be calculated with equations [31].

#### Information on use of health care

We will use the available databases from two health care insurers to obtain data on health care use of the participants. These two companies have confirmed their cooperation.

### Sample Size Calculation

Input for the sample size calculation is derived from a study performed amongst Surinamese (Hindustani and Creole Surinamese) people in the Netherlands (SUNSET study) [10]. The amount of physical activity in this group of women (n = 666) was measured with the SQUASH [26]. From the SUNSET study it appeared that Surinamese women were on the average 1852 (SD: 1100) minutes per week physically active. To be able to detect an absolute difference of 15% in the number of minutes of physical activity per week with an one-sided alpha of 0.05, and a power of 80% 180 women in both groups are needed.

### Data Analyses

The main aim of the study is to asses the effect of the intervention on physical activity. Therefore group differences between groups and 95% confidence intervals will be calculated in changes in physical activity after the intervention (between T0 and T1) and in maintenance (T0-T2). The statistical analysis will be performed according to the intention-to-treat principle.

Between group differences will be calculated using the Student t-test for continuous variables or Chi-Square for dichotomous variables. Baseline comparability will be investigated by descriptive statistics to examine whether randomisation was successful. If necessary, adjustments for baseline variables will be performed in the analysis.

Finally, multivariate regression analysis will be conducted to examine the influence of baseline variables and the degree of participation in the intervention on the outcome.

Within the intervention group multivariate regression analysis will be conducted to examine the influence of baseline variables and the degree of participation in the intervention (i.e. the proportion of sessions attended and the components of the intervention followed) on the changes in physical activity.

## Discussion

This trial will provide information on the effectiveness of an exercise referral scheme (ERS) on the short and long term among women from ethnic minority groups in the Netherlands. When effective, the intervention may play an important role in the promotion of physical activity among women from ethnic minority groups.

ERS seem particularly useful for those groups who are most in need of supervised exercise. Women from ethnic minority groups living in deprived neighborhoods might be considered as one of those groups. They experience a poorer health compared to ethnic Dutch women and are more often physically inactive [9]. For these women, participating in ERS might be a first step towards better health. The present trial will be the first assessing the effect of an exercise referral scheme on physical activity among women with an ethnic minority background

Besides measuring physical activity we also assess, in contrast to most other studies, a broader range of health aspects (e.g. well-being, fitness, perceived health and motivational factors). It might be that level of physical activity of these women does not improve significantly following the exercise programme, but that it may improve their health status and their intention (and motivation) for being physically active. Considering the poor health status of women from ethnic minority groups, these are important outcomes as well.

The study will measure the effect of the programme not only after completion, but also six months after completion of the exercise sessions. The importance of long term effects is stressed by many evaluation studies. With two follow-up measurements we hope to provide insight into effects over a longer period of time.

With the results of this study we will contribute to the evidence base for physical activity interventions among ethnic minority populations.

## Abbreviations

ERS: Exercise referral schemes; GP: General practitioner; EoP: Exercise on Prescription.

## Competing interests

The authors declare that they have no competing interests.

## Authors' contributions

KH and MD are the main investigators and contributed equally to the manuscript. KH, MD and KS are responsible for general study design. MD planned the statistical analysis, and conducted the sample size calculation. All authors were responsible for the drafting of this paper and approved the final manuscript

## Pre-publication history

The pre-publication history for this paper can be accessed here:


